# Multi-regional debridement and staged reconstruction for fracture-related infection after intramedullary nailing

**DOI:** 10.5194/jbji-11-655-2026

**Published:** 2026-10-29

**Authors:** Xingyu Chen, Junyi Li, Yunan Li, Hangyu Li, Kehan Lv, Yueliang Zhu, Muguo Song, Jian Shi

**Affiliations:** 1 Department of Orthopaedics, 920th Hospital of the Joint Logistics Support Force of the PLA, 212 Daguan Road, Kunming, 650032, China; 2 Yunnan Province Key Laboratory of Microsurgical Medical Technology, 212 Daguan Road, Kunming 650032, China; 3 Department of Orthopaedics, The Second Affiliated Hospital, Zhejiang University School of Medicine, Hangzhou, China

## Abstract

Late fracture-related infection (FRI) after intramedullary nailing may extend beyond the fracture site to involve the medullary canal, nail entry site, and locking screw tracts. This retrospective cohort study evaluated a staged protocol incorporating systematic multi-regional debridement of the entire nail-related infection pathway.

We reviewed 47 patients treated between January 2017 and January 2025, all of whom had fracture nonunion and an interval of at least 3 months from the first documented confirmation of FRI to admission. Stage I included intramedullary nail removal; systematic multi-regional debridement of the fracture/nonunion site, nail entry site, entire medullary canal, and proximal and distal locking screw tracts; locking plate fixation; and temporary vancomycin-loaded polymethyl methacrylate cement placement for dead-space management and induced membrane formation. Definitive Stage II reconstruction included the removal of the temporary plate construct and cement, repeat debridement, bone grafting, and definitive fixation with a new internal locking plate after clinical and laboratory infection quiescence and intraoperative reassessment.

The cohort included 21 tibial and 26 femoral cases, with a mean follow-up of 25.3 ± 14.1 months. Two patients had confirmed persistent infection requiring repeat debridement before definitive Stage II reconstruction. No confirmed recurrence or unplanned surgical debridement for suspected recurrence occurred after definitive reconstruction. Union after the index Stage II reconstruction was achieved in 44 of 47 patients (93.6 %). The remaining three patients achieved union after further surgery, resulting in a final union rate of 100 %. The mean times to radiographic union and full weight-bearing were 9.7 ± 2.1 and 11.3 ± 3.8 months, respectively. Median (interquartile range) WBC count, ESR, and CRP decreased from 7.2 (6.2–9.0) × 10^9^ L^−1^, 39.0 (12.5–65.0) mm h^−1^, and 26.5 (10.3–43.2) mg L^−1^ to 5.9 (5.0–7.7) × 10^9^ L^−1^, 17.0 (8.0–29.5) mm h^−1^, and 6.3 (3.7–8.9) mg L^−1^, respectively. LEFS and SF-36 scores increased from 47.0 (37.0–65.5) and 417.0 (335.0–439.0) to 70.0 (61.0–75.5) and 491.0 (480.0–516.5), whereas VAS and SAS scores decreased from 4.0 (4.0–4.0) and 45.0 (42.0–50.0) to 1.0 (0.0–1.0) and 32.0 (29.5–37.0), respectively (all p≤0 .001). These findings support the feasibility of this staged protocol in selected patients with late post-intramedullary-nailing FRI and nonunion.

## 1 Introduction

Fracture-related infection (FRI) is a serious complication of fracture fixation and is associated with prolonged treatment, repeated surgery, impaired fracture healing, functional loss, and substantial healthcare burden (Metsemakers et al., 2024; Rupp et al., 2024; Depypere et al., 2020b). Management is particularly challenging when infection is accompanied by nonunion, bone necrosis, implant-related biofilm, soft-tissue compromise, or bone defects (Simpson and Tsang, 2017; Moriarty et al., 2022; Depypere et al., 2020b). In such cases, treatment must address not only infection control but also restoration of stability, dead-space management, soft-tissue readiness, and bone reconstruction.

Intramedullary nailing is widely used for tibial and femoral shaft fractures because of its mechanical stability, relatively limited soft-tissue disruption, and high rates of fracture union (Wood, 2006; Duan et al., 2012; Egol et al., 2007). Although infection after intramedullary nailing is relatively uncommon (Young et al., 2011; Court-Brown, 2004), late post-intramedullary nailing FRI represents a distinct clinical problem. Pathogens may spread along the nail and endosteal surface, and infection foci may involve not only the fracture site but also the nail entry site, medullary canal, and proximal and distal locking screw tracts (Fig. 1a) (Makridis et al., 2013; Zhang et al., 2020; Oliveira et al., 2023). Therefore, debridement limited to the fracture site may leave residual infection within the medullary canal or around the locking screw tracts. For late infection with nonunion or suspected mature biofilm formation, implant retention alone is unlikely to achieve adequate infection control; implant removal or exchange, debridement, local antimicrobial treatment, and systemic antibiotics are usually required (Baertl et al., 2024; Metsemakers et al., 2020; Makridis et al., 2013; Zhang et al., 2020; Oliveira et al., 2023; Depypere et al., 2020a).

In this study, the anatomical distribution of post-intramedullary nailing infection was defined as potential involvement of the fracture site, nail entry site, medullary canal, and proximal and distal locking screw tracts. We developed a staged protocol combining intramedullary nail removal, multi-regional debridement of these anatomical regions, locking plate fixation, temporary vancomycin-loaded polymethyl methacrylate cement for dead-space management and induced membrane formation, and subsequent bone grafting with definitive fixation. The reconstructive component followed the principles of the Masquelet induced membrane technique, whereas the distinguishing feature of the present protocol was systematic debridement of the entire nail-related infection pathway rather than debridement limited to the fracture site (Masquelet et al., 2000). This study aimed to evaluate the clinical outcomes of this protocol.

**Figure 1:**
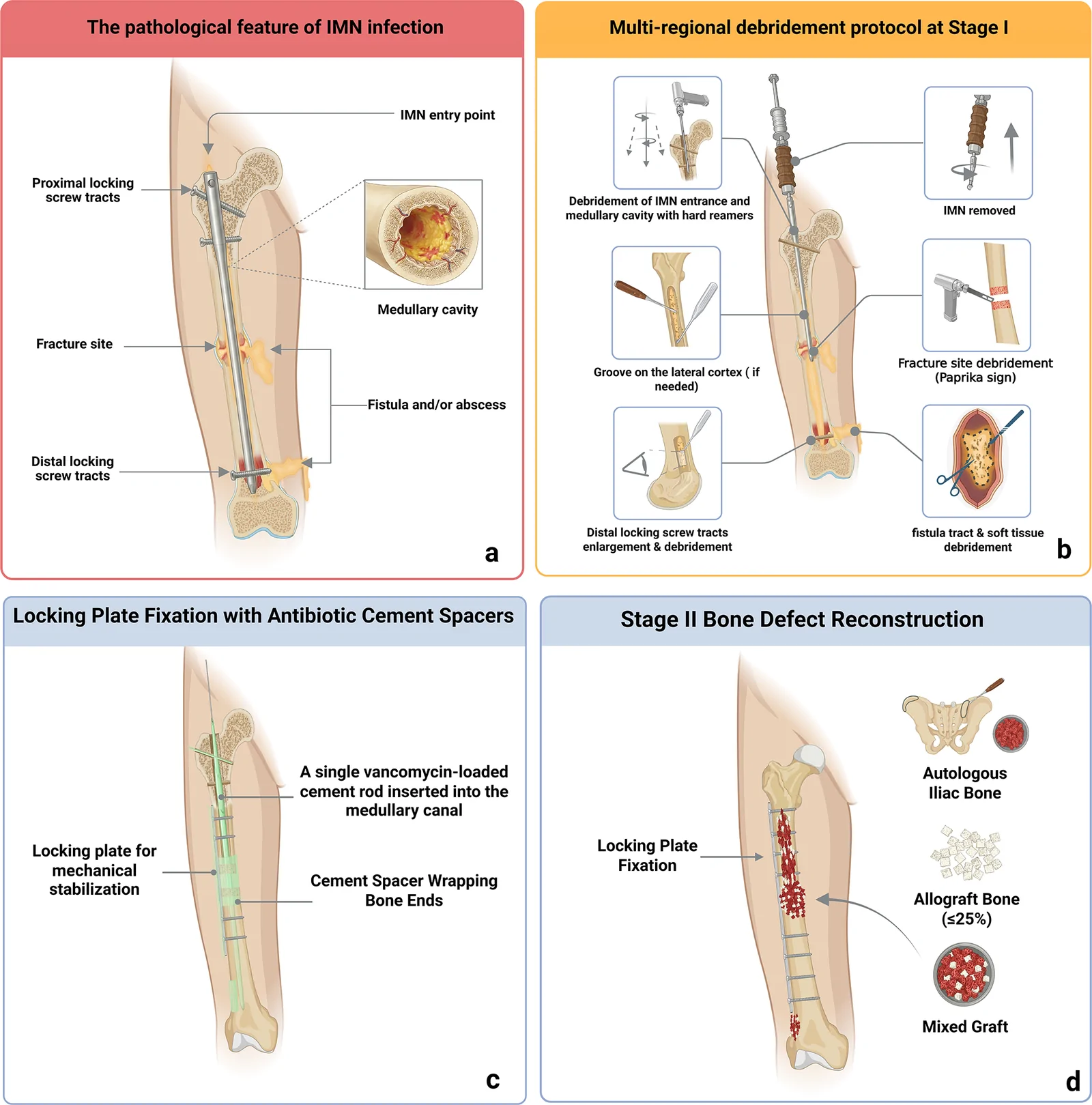
Anatomical distribution and staged treatment concept for late post-intramedullary nailing fracture-related infection. **(a)** Potential nail-related infection regions. **(b)** Anatomical targets of Stage I multi-regional debridement. **(c)** Locking plate fixation with non-load-bearing vancomycin-loaded cement rods and spacers for local antimicrobial delivery, dead-space management, and induced membrane formation. **(d)** Stage II bone grafting and definitive fixation performed only when residual infection was not suspected. Created with https://BioRender.com (last access: 28 October 2026).

## 2 Patients and methods

### 2.1 Study design and patients

This retrospective cohort study included consecutive patients treated at a tertiary referral centre between January 2017 and January 2025. Eligible patients had late FRI after tibial or femoral intramedullary nailing and were treated using a staged reconstruction protocol based on multi-regional debridement and fixation replacement.

FRI was diagnosed using the international consensus framework, integrating clinical, microbiological, and histopathological evidence (Metsemakers et al., 2018). Clinical confirmatory findings included either a sinus tract or wound breakdown communicating with bone or implant, or purulent drainage or intraoperative pus. Microbiological confirmation required phenotypically indistinguishable microorganisms isolated from at least two separate deep specimens. Histopathological reports were reviewed in all patients. In late-onset cases, histopathological confirmation was defined as the presence of more than five polymorphonuclear neutrophils per high-power field in at least five separate high-power fields (Govaert et al., 2020). For time-to-onset classification, the interval from fracture fixation to the first documented onset of infection-related symptoms or signs was categorized according to the Willenegger–Roth framework as early onset ( < 2 weeks), delayed onset (3–10 weeks), or late onset ( > 10 weeks) (Metsemakers et al., 2020; Willenegger and Roth, 1986). Separately, for eligibility and descriptive analyses, the documented FRI interval was defined as the time from the earliest documented confirmation of FRI to admission for Stage I treatment at our centre. Patients were included if they met the following criteria: (1) previous tibial or femoral fracture treated with intramedullary nailing; (2) FRI diagnosed according to the criteria described above, with a documented FRI interval of at least 3 months before admission; (3) fracture nonunion; (4) treatment with the staged protocol described below; and (5) age between 18 and 60 years. Exclusion criteria were Cierny–Mader physiological class C host status, severe immunodeficiency or uncontrolled autoimmune disease precluding staged reconstruction, and follow-up of less than 12 months.

The study was conducted in accordance with the Declaration of Helsinki and was approved by the Medical Ethics Committee of the 920th Hospital of the Joint Logistics Support Force of the PLA. Informed consent was obtained from all participants.

### 2.2 Definitions and outcomes

The primary outcome was confirmed infection recurrence after definitive Stage II reconstruction. Confirmed recurrence required at least one confirmatory FRI criterion: a sinus tract or wound breakdown communicating with bone or implant, purulent drainage or intraoperative pus, phenotypically indistinguishable microorganisms isolated from at least two separate deep specimens, or fulfilment of the predefined histopathological criterion (Metsemakers et al., 2018). Clinical concern for recurrent infection without fulfilment of a confirmatory criterion was recorded separately as suspected recurrence. Any unplanned surgical debridement for suspected or confirmed infection after definitive Stage II reconstruction was classified as post-reconstruction treatment failure.

Before definitive Stage II reconstruction, confirmed persistent infection was defined using the same FRI confirmatory criteria. Intraoperative warning findings that prompted additional sampling, repeat debridement, and postponement of bone grafting but did not fulfil a confirmatory criterion were classified as suspected residual infection. Confirmed persistent infection and suspected residual infection before definitive reconstruction were recorded as interstage treatment failures and were not counted as post-reconstruction recurrence.

Secondary outcomes included repeat debridement before final infection control, radiographic bone union, time to full weight-bearing, complications, inflammatory markers, and functional outcomes. Bone union was defined as bridging callus across at least three of four cortices on anteroposterior and lateral radiographs, together with the absence of pain or abnormal motion at the reconstruction site (Corrales et al., 2008). Time to full weight-bearing was defined as the interval from definitive Stage II reconstruction to the first documented follow-up visit at which unrestricted full weight-bearing was permitted by the treating surgeon. Inflammatory markers included white blood cell count, erythrocyte sedimentation rate, and C-reactive protein. These markers were evaluated as non-specific longitudinal adjuncts rather than as stand-alone tests to confirm or exclude infection. Functional outcomes included the Visual Analogue Scale (VAS; range 0–10, with higher scores indicating greater pain), Lower Extremity Functional Scale (LEFS; range 0–80, with higher scores indicating better lower-limb function), 20-item Self-Rating Anxiety Scale (SAS) raw score (range 20–80, with higher scores indicating more severe anxiety symptoms), and Short Form-36 (SF-36) total score (calculated as the sum of the eight domain scores, range 0–800, with higher scores indicating better health-related quality of life).

### 2.3 Operative protocol

All patients were treated using a two-stage protocol. In Stage I, the intramedullary nail was removed, and systematic multi-regional debridement was performed in all patients. The fracture/nonunion site, nail entry site, entire medullary canal, and proximal and distal locking screw tracts were routinely treated as components of the potential nail-related infection pathway (Fig. 1b). Necrotic soft tissue, fistula tracts, sequestra, inflammatory granulation tissue, and purulent material were removed. The medullary canal was sequentially reamed using the diameter of the removed intramedullary nail as a reference. The final reaming diameter was individualized according to medullary canal morphology and cortical integrity but was at least 2 mm larger than the removed nail diameter. All proximal and distal locking screw tracts were routinely inspected and debrided along their entire trajectories. A tract was enlarged when gross loosening of the corresponding locking screw, visible inflammatory granulation tissue, purulence, or other macroscopic evidence of infection was present. Enlargement was performed using a drill bit with a diameter larger than that of the removed locking screw. If drilling alone did not adequately remove the involved tissue, the tract was additionally curetted. Irrigation and negative-pressure suction were performed through the treated tract to remove residual debris and permit inspection of the tract and adjacent medullary canal. Debridement was continued until no visible inflammatory tissue, purulence, or loose debris remained along the tract. No dedicated continuous irrigation-aspiration system or pressurized lavage device was used. After reaming, sterile normal saline was manually injected into the medullary canal through a standard sterile suction tube using a syringe, without simultaneous negative-pressure suction. After each irrigation cycle, the suction tube was introduced through the treated distal locking screw tract and connected to negative-pressure suction to remove residual fluid and debris. The canal was then reinspected. Reaming, curettage, irrigation, and intermittent suction were repeated as required until no visible inflammatory granulation tissue or purulent material remained and the irrigation effluent was clear of macroscopic debris. Additional cortical windows or longitudinal grooves were created in 14 patients when the adequacy of intramedullary debridement could not be confirmed through the nail entry site and locking screw tracts alone.

After debridement, mechanical stability was restored using locking plate fixation. All femoral cases received internal plate fixation. In tibial cases, internal or externalized plate fixation was selected according to the local soft-tissue condition. Screw placement across areas of gross purulence, necrotic bone, or the planned grafting zone was avoided whenever possible. Polymethyl methacrylate cement was manually mixed with vancomycin at a fixed concentration of 5 g per 40 g of cement powder. The vancomycin-loaded cement was used for dead-space management, local Gram-positive antimicrobial delivery, defect filling, and induced membrane formation (Fig. 1c) (Metsemakers et al., 2019). The cement rods and defect-filling cement were non-load-bearing local antibiotic carriers and spacers rather than cement-coated intramedullary fixation devices; mechanical stability was provided by the locking plate construct. The intramedullary cement rod was manually moulded around either a single Kirschner wire (K-wire) or two K-wires twisted together. The diameter of the cement rod did not exceed that of the removed intramedullary nail. The K-wire core extended beyond the cement mantle at the insertion end to facilitate subsequent removal. A single continuous cement rod was introduced through the original nail entry site and advanced distally within the medullary canal. Additional small cement rods were placed in the enlarged locking screw tracts. For large-diameter proximal fixation tracts associated with cephalomedullary nails, the tract-specific cement rod was likewise moulded around a K-wire core. At Stage II, the intramedullary cement rod was withdrawn through the original nail entry site, whereas the tract-specific cement rods were removed through their respective tracts. The remaining cement was used to fill the bone defect and cover the fixation construct when appropriate. Soft-tissue defects were managed with flap coverage when primary closure was not feasible.

Definitive Stage II reconstruction was not performed at a fixed time point. Patients were scheduled for a planned Stage II procedure no earlier than 6 to 8 weeks after Stage I and only after clinical and laboratory infection quiescence. The preoperative criteria included healing of the incision or fistula tract; absence of local signs of infection; and normalization or near normalization of white blood cell count, erythrocyte sedimentation rate, and C-reactive protein on repeated assessments. No single serum inflammatory-marker cutoff was used in isolation; results were interpreted according to serial trends and institutional reference ranges together with wound healing and soft-tissue findings (Metsemakers et al., 2018, 2020).

At the planned Stage II procedure, the Stage I locking plate and all vancomycin-loaded cement rods and spacers were removed. Repeat debridement was performed, and the previous screw tracts were inspected, curetted, and irrigated. The operative field was reassessed before bone grafting. Purulence was regarded as confirmatory evidence of active infection (Metsemakers et al., 2018), whereas unexpected turbid or excessive fluid accumulation and abnormal inflammatory granulation tissue of questionable quality or viability were treated as intraoperative warning findings.

When these findings raised concern for residual infection, multiple deep-tissue specimens were obtained for microbiological culture, and intraoperative frozen-section histopathological examination was performed. The decision to defer bone grafting and perform further debridement was based on the macroscopic findings and was made irrespective of the frozen-section result. When the decision to defer reconstruction was made after removal of the Stage I plate, temporary plate fixation was re-established to maintain mechanical stability during the additional infection-control interval. Definitive reconstruction was rescheduled after clinical and laboratory infection quiescence and soft-tissue readiness were re-established (Metsemakers et al., 2020).

When definitive reconstruction proceeded, the existing temporary plate construct was removed, bone grafting was performed, and definitive stability was restored using a new internal locking plate. No intramedullary nail was reinserted (Fig. 1d). Autologous iliac cancellous bone was used for grafting. If the autologous graft volume was insufficient, morselized allograft was added as an extender, with the allograft proportion limited to no more than 25 % according to the institutional protocol (Pesciallo et al., 2021).

### 2.4 Microbiological procedures and antimicrobial management

Whenever clinically feasible, systemic antibiotics were discontinued for 1 to 2 weeks before Stage I surgery to improve microbiological yield. In two patients with systemic sepsis or rapidly progressive infection, antibiotics were continued and no preoperative antibiotic-free interval was used. At Stage I, 4.34 ± 0.96 deep specimens were obtained per patient before empirical postoperative antimicrobial therapy was initiated; the median number was 5 (interquartile range 4–5; range 2–5). Sampling included deep soft tissue and at least one lesional bone specimen in all patients. Multi-site deep soft-tissue sampling was performed in 16 patients. Representative sampling sites included the fracture site, medullary canal, nail entry site, and locking screw tracts.

Each specimen was placed in a separate sterile container and promptly transported to the microbiology laboratory. The specimens were inoculated onto agar media and incubated in a microbiological incubator. Separate instruments were not routinely used for each specimen. Aerobic cultures were incubated for 5 d. In cases without aerobic growth, anaerobic cultures were maintained for 14 d. Molecular testing was not routinely performed. Histopathological examination was performed in all patients. Additional details are provided in Table S3 in the Supplement.

Pending culture results, empirical intravenous treatment consisted of ceftriaxone 2 g once daily. Treatment was subsequently adjusted according to the identified organism and susceptibility profile. All patients received a total of 42 d of systemic antimicrobial therapy after Stage I, consisting of 14 d of initial intravenous treatment followed by 28 d of susceptibility-directed continuation therapy. Continuation therapy was administered orally when an active oral agent was available and intravenously when no active oral option was available. In culture-positive cases, antimicrobial agents were selected according to the identified organism and susceptibility profile. In culture-negative cases, the institutional regimen consisted of intravenous ceftriaxone 2 g once daily for 14 d, followed by oral levofloxacin 500 mg once daily plus rifampicin 450 mg once daily for 28 d. This regimen reflected institutional practice during the study period. The antimicrobial strategies are summarized in Table S1 in the Supplement.

Whenever repeat debridement was required before definitive Stage II reconstruction, multiple deep-tissue specimens were obtained before antimicrobial treatment was restarted. After repeat debridement, both affected patients began a new 6-week systemic antimicrobial course consisting of 14 d of intravenous treatment followed by 28 d of oral treatment. Patients who proceeded to definitive Stage II reconstruction without evidence of active infection received routine surgical prophylaxis with a single pre-incision dose of cefazolin; no postoperative therapeutic antimicrobial course was administered.

Vancomycin-loaded cement was regarded as an adjunct to debridement, dead-space management, local antimicrobial delivery, and systemic antimicrobial therapy, not as a stand-alone antimicrobial treatment (Metsemakers et al., 2019; Pesciallo et al., 2021; Depypere et al., 2020a).

### 2.5 Follow-up

Patients were followed monthly during the first 3 months after definitive Stage II reconstruction, every 3 months during the first postoperative year, every 6 months during the second year, and annually thereafter. At each follow-up visit, clinical signs of infection recurrence were assessed, including redness, swelling, increasing pain, wound drainage, or new sinus tract formation. White blood cell count, erythrocyte sedimentation rate, and C-reactive protein were reviewed. Radiographs were obtained to evaluate bone union. Functional scores were assessed at initial admission and final follow-up.

### 2.6 Statistical analysis

Statistical analyses were performed using IBM SPSS Statistics version 25.0. Continuous variables were assessed for normality using the Shapiro–Wilk test. Normally distributed variables are presented as mean and standard deviation, and non-normally distributed variables as median and interquartile range. Categorical variables are presented as numbers; percentages are provided only when clinically informative. Paired comparisons between initial admission and final follow-up were performed using the paired-samples t -test or Wilcoxon signed-rank test, as appropriate. A two-sided p value < 0.05 was considered statistically significant.

## 3 Results

### 3.1 Patient characteristics and microbiology

A total of 47 consecutive patients with late FRI after intramedullary nailing were included. There were 39 male and eight female patients, with a mean age of 40.2 ± 11.4 years. The mean documented FRI interval from the earliest documented confirmation of FRI to admission was 15.3 ± 15.7 months. All patients had a documented FRI interval of at least 3 months and fracture nonunion. Based on the interval from fracture fixation to the first documented onset of infection-related symptoms or signs, all infections were classified as late-onset FRI. The infected bone was the tibia in 21 patients and the femur in 26 patients. Eight patients had open fractures and 39 had closed fractures. Recorded comorbidities or risk factors included diabetes mellitus in nine patients, smoking in 17, immunosuppression or glucocorticoid use in three, and deep vein thrombosis in two (Table 1).

**Table 1:** Preoperative characteristics of the patients ( n=47 ).

Characteristic	Value
Sex	
Male, n	39
Female, n	8
Age, years, mean ± SD	40.2 ± 11.4
Age range, years	18–58
Duration of infection, months, mean ± SD	15.3 ± 15.7
Duration range, months	3–96
Fracture type, n	
Closed fracture	39
Open fracture	8
Location, n	
Tibia	21
Femur	26
Comorbidity or risk factor, n	
None	16
Diabetes mellitus	9
Smoking	17
Immunosuppression/glucocorticoid use	3
Deep vein thrombosis	2

Histopathological examination was performed in all 47 patients, and all pathology reports described changes compatible with osteomyelitis. Of the 17 culture-negative patients, 15 fulfilled clinical confirmatory criteria based on a communicating sinus and/or intraoperative purulence. The remaining two patients fulfilled the histopathological confirmatory criterion, with more than five polymorphonuclear neutrophils per high-power field identified in at least five separate high-power fields. Deep-tissue cultures were positive in 30 patients and negative in 17. The distribution of identified organisms and multidrug resistance is summarized in Table 2.

**Table 2:** Deep-tissue culture results and multidrug resistance among 47 patients.

Microbiological category	n	Multidrug
		resistance
Culture negative	17	–
Gram-positive bacteria	18	–
MSSA	11	0
MRSA	4	4
*Enterococcus faecalis*	3	0
Gram-negative bacteria	12	–
*Acinetobacter baumannii*	3	0
*Pseudomonas aeruginosa*	3	2
*Escherichia coli*	4	0
*Klebsiella pneumoniae*	2	1

Note: MDR was defined as acquired non-susceptibility to at least one agent in three or more antimicrobial categories (Magiorakos et al., 2012). MSSA, methicillin-sensitive *Staphylococcus aureus*; MRSA, methicillin-resistant *Staphylococcus aureus*; MDR, multidrug resistance.

### 3.2 Surgical course

After debridement, 21 patients had segmental bone defects and 26 had local bone defects. The mean segmental defect length was 6.7 cm, with a range of 4 to 13 cm. All patients were stabilized with locking plate constructs after Stage I debridement, including internal plate fixation in 37 patients and externalized plate fixation in 10. Additional cortical windows or longitudinal grooves were required in 14 patients. Flap coverage was required in four patients.

The mean interval between Stage I and definitive Stage II reconstruction was 11.4 weeks (range 7–22 weeks). At definitive Stage II reconstruction, the existing temporary plate construct was removed and replaced with a new internal locking plate in all 47 patients; no intramedullary nail was reinserted. Reconstruction was not performed at a fixed interval; timing was determined by wound healing, the absence of local signs of infection, inflammatory-marker trends, soft-tissue readiness, and the need for repeat debridement.

Two patients required repeat debridement before definitive Stage II reconstruction. One patient developed fever, wound drainage, and elevated serum inflammatory markers during the interstage period. At repeat debridement, five deep specimens were obtained, four of which yielded phenotypically indistinguishable *Staphylococcus aureus*. Histopathological examination demonstrated dense neutrophilic infiltration. This event fulfilled microbiological confirmatory criteria and was classified as confirmed persistent infection before definitive Stage II reconstruction.

The other patient had no preoperative clinical or laboratory evidence of active infection. At the planned Stage II procedure, removal of the Stage I plate and cement spacer revealed unexpected turbid or excessive fluid accumulation and abnormal inflammatory granulation tissue in the absence of purulence. On the basis of these macroscopic findings, bone grafting was deferred immediately, repeat debridement was performed, and temporary plate fixation was re-established to maintain mechanical stability during the additional infection-control interval. Multiple deep-tissue specimens were obtained for microbiological culture, and an intraoperative frozen-section histopathological examination was performed, with the result available during surgery. Five deep specimens remained culture negative; however, final histopathological examination demonstrated more than five polymorphonuclear neutrophils per high-power field in at least five separate high-power fields. The event therefore fulfilled the histopathological confirmatory criterion and was classified as confirmed persistent infection.

Both patients subsequently completed a new 42 d systemic antimicrobial course and met the predefined infection-quiescence criteria. They then underwent definitive Stage II reconstruction. In the second patient, the interim plate was removed and replaced with a new internal locking plate at the rescheduled procedure.

A representative case showing preoperative sinus formation, infected nonunion, Stage I multi-regional debridement, Stage II reconstruction, radiographic union, and final functional recovery are shown in Fig. 2.

**Figure 2:**
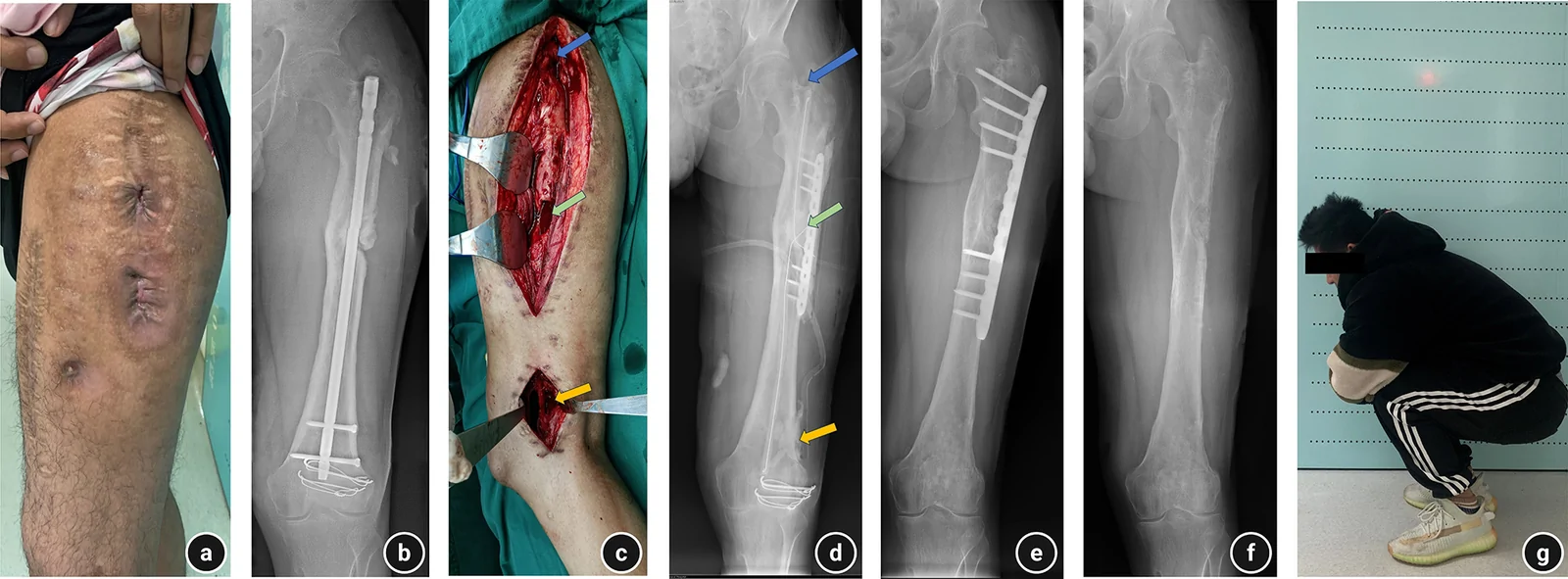
Representative case of late fracture-related infection after femoral intramedullary nailing. **(a)** Preoperative photograph showing sinus tracts and scarred soft tissue. **(b)** Preoperative radiograph showing infected femoral nonunion after intramedullary nailing. **(c)** Intraoperative view after nail removal and multi-regional debridement. The blue, green, and yellow arrows indicate the proximal nail entry region, fracture/nonunion site, and distal locking screw tract, respectively. **(d)** Stage I radiograph showing temporary locking plate fixation with non-load-bearing vancomycin-loaded polymethyl methacrylate cement rods and spacers for local antimicrobial delivery and dead-space management. **(e)** Stage II radiograph after removal of the Stage I plate and cement, bone grafting, and definitive fixation with a new internal locking plate. **(f)** Final follow-up radiograph showing bone union and remodelling after implant removal. **(g)** Final follow-up photograph showing functional recovery.

### 3.3 Infection control, union, and complications

The mean follow-up after definitive Stage II reconstruction was 25.3 ± 14.1 months, with a range of 12 to 52 months. No confirmed infection recurrence or unplanned surgical debridement for suspected recurrence occurred after definitive Stage II reconstruction. Outcomes stratified by microbiological category are shown in Table S2 in the Supplement.

Union after the index Stage II reconstruction was achieved in 44 of 47 patients (93.6 %). Three patients, including two femoral cases and one tibial case, had persistent nonunion and underwent repeat bone grafting with or without fixation revision. All three subsequently achieved union, resulting in a final union rate of 100 %. The mean time to radiographic union was 9.7 ± 2.1 months, with a range of 5 to 11 months. Full weight-bearing was achieved at a mean of 11.3 ± 3.8 months, with a range of 8 to 17 months. Delayed iliac donor-site wound healing occurred in five patients; all events were superficial and resolved with local wound care without surgical re-intervention. The specific causes of delayed healing could not be reliably determined from the available retrospective medical records. Postoperative knee stiffness occurred in five patients with femoral infection. Limb length discrepancy occurred in three patients, measuring 2.7, 3.0, and 4.3 cm.

### 3.4 Laboratory and functional outcomes

WBC count, ESR, and CRP decreased; LEFS and SF-36 scores increased; and VAS and SAS scores decreased between initial admission and final follow-up (WBC, p=0 .001; all other comparisons, p<0 .001; Table 3).

**Table 3:** Laboratory and functional outcomes from initial admission to final follow-up, n=47 .

Variable	Initial admission	Final follow-up	Change	P value
LEFS score	47.0 (37.0, 65.5)	70.0 (61.0, 75.5)	14.0 (7.5, 28.5)	< 0.001
VAS score	4.0 (4.0, 4.0)	1.0 (0.0, 1.0)	- 3.0 ( - 4.0, - 3.0)	< 0.001
SF-36 score	417.0 (335.0, 439.0)	491.0 (480.0, 516.5)	93.0 (55.5, 144.0)	< 0.001
SAS score	45.0 (42.0, 50.0)	32.0 (29.5, 37.0)	- 14.0 ( - 18.0, - 7.5)	< 0.001
WBC, × 10^9^ L^−1^	7.2 (6.2, 9.0)	5.9 (5.0, 7.7)	- 0.9 ( - 2.9, 0.0)	0.001
ESR, mm h^−1^	39.0 (12.5, 65.0)	17.0 (8.0, 29.5)	- 16.0 ( - 44.0, 0.5)	< 0.001
CRP, mg L^−1^	26.5 (10.3, 43.2)	6.3 (3.7, 8.9)	- 21.7 ( - 36.0, - 4.0)	< 0.001

Note: values are presented as median (interquartile range). Changes represent paired within-patient differences.

## 4 Discussion

This study evaluated a staged treatment protocol for late FRI after tibial or femoral intramedullary nailing. Two patients had confirmed persistent infection requiring repeat debridement before definitive Stage II reconstruction, whereas no confirmed recurrence occurred thereafter during the available follow-up. Union was achieved after the index Stage II reconstruction in 44 of 47 patients (93.6 %) and after further surgery in the remaining three. These findings support the feasibility of the protocol, although the absence of a comparator group precludes conclusions about superiority over other treatment strategies.

The reconstructive component of the protocol followed the principles of the Masquelet induced membrane technique, whereas its principal distinction was systematic debridement of the entire nail-related infection pathway rather than treatment of the fracture site alone. Infection may extend along the implant and endosteal surface beyond the fracture site, providing the rationale for systematic debridement of the entire nail-related infection pathway (Metsemakers et al., 2020; Makridis et al., 2013; Zhang et al., 2020; Oliveira et al., 2023; Pesciallo et al., 2021). Mature biofilm, necrotic bone, and nonunion in late infection further increase the likelihood that infection persists beyond the immediately visible fracture site (Simpson and Tsang, 2017; Moriarty et al., 2022; Depypere et al., 2020b). Recent studies have demonstrated the heterogeneity of management after intramedullary-nailing FRI, including antimicrobial suppression to facilitate fracture union and both nail-retention and nail-removal strategies. In contrast, the present cohort was restricted to late FRI with nonunion and was treated with nail removal and staged reconstruction (Tsang et al., 2024; Schiefer et al., 2026).

Mechanical stability, dead-space management, soft-tissue management, and staged bone reconstruction were complementary components of the treatment strategy. FRI treatment requires coordinated surgical control, systemic antimicrobial therapy, stabilization, and optimization of the local and systemic host environment (Li et al., 2024; Metsemakers et al., 2019, 2020). Locking plate fixation provided mechanical stability after intramedullary nail removal and debridement, whereas vancomycin-loaded polymethyl methacrylate cement served as a non-load-bearing local antimicrobial carrier, dead-space filler, and induced-membrane spacer (Bose et al., 2015; McNally et al., 1993; Metsemakers et al., 2019). Stage II reconstruction was not performed at a fixed interval; serial clinical assessment, inflammatory-marker trends, soft-tissue readiness, and intraoperative reassessment were considered together. Suspicious intraoperative findings prompted additional sampling, repeat debridement, and postponement of bone grafting rather than being regarded as independent confirmatory criteria (Metsemakers et al., 2018, 2020).

Because vancomycin primarily targets Gram-positive organisms and does not provide reliable coverage for the Gram-negative pathogens identified in this cohort, the observed outcomes should be attributed to the combined protocol of complete nail removal, multi-regional debridement, restoration of stability, dead-space and soft-tissue management, and culture-directed systemic antimicrobial therapy rather than to local vancomycin delivery alone (Metsemakers et al., 2019; Depypere et al., 2020a). Further research may compare alternative local antimicrobial combinations, absorbable carriers, and antimicrobial-coated fixation devices in appropriately selected post-intramedullary-nailing FRI populations (Anagnostakos et al., 2009; Pedroni et al., 2024; Walter et al., 2022; Jacob et al., 2023).

Diagnosis in this retrospective cohort relied on clinical confirmatory findings, deep-tissue microbiology, histopathology, conventional imaging, and intraoperative assessment. These components provided complementary information but were not applied uniformly in every patient. Serum WBC count, ESR, and CRP were used as non-specific longitudinal adjuncts rather than stand-alone diagnostic tests, and advanced nuclear or hybrid imaging was not systematically performed. Consequently, the sensitivity of preoperative detection and delineation of the complete anatomical extent of infection may have varied among patients (Metsemakers et al., 2018, 2020).

This study has several limitations. First, it was a single-centre retrospective cohort without a comparator group and was therefore subject to selection bias, residual confounding, and indication bias. Some potentially relevant host factors were not systematically available. Second, the sample size was limited, and subgroup findings should be interpreted as descriptive. Third, Stage I microbiological procedures were not fully standardized: four patients had only two deep specimens, separate instruments were not routinely used for each specimen, aerobic incubation was limited to 5 d, and anaerobic culture was performed selectively. Conventional agar-based culture was used; however, the specific agar formulations, incubation conditions, and detailed specimen-transport procedures were not consistently documented and could not be reliably reconstructed from the retrospective records. These factors may have contributed to the proportion of culture-negative cases. Fourth, the timing of Stage II was individualized, and microbiological sampling at the planned Stage II procedure was selective rather than systematic. Therefore, subclinical residual infection could not be excluded in all patients, and the absence of clinical recurrence should not be interpreted as culture-confirmed eradication. Finally, the available follow-up may have been insufficient to detect all late recurrences.

## 5 Conclusions

This single-centre retrospective cohort supports the feasibility of a staged protocol combining intramedullary nail removal, systematic multi-regional debridement of the entire nail-related infection pathway, temporary locking plate fixation, dead-space management, and subsequent bone grafting with new internal plate fixation for late fracture-related infection with nonunion. However, the absence of a comparator group, non-standardized microbiological procedures, and limited follow-up preclude conclusions about superiority or definitive infection eradication. Further multicentre comparative studies are required.

## Supplement

10.5194/jbji-11-655-2026-supplement The supplement related to this article is available online at https://doi.org/10.5194/jbji-11-655-2026-supplement.

## Data Availability

The de-identified clinical data supporting the findings of this study are available from the corresponding author upon reasonable request, subject to institutional approval and a data use agreement. Individual patient-level data are not publicly available because of privacy and ethical restrictions.

## Appendix A Abbreviations used in this article

**Table:**

FRI	fracture-related infection
WBC	white blood cell
ESR	erythrocyte sedimentation rate
CRP	C-reactive protein
VAS	visual analogue scale
LEFS	Lower Extremity Functional Scale
SAS	Self-Rating Anxiety Scale
SF-36	Short Form-36
MSSA	methicillin-sensitive *Staphylococcus aureus*
MRSA	methicillin-resistant *Staphylococcus aureus*
MDR	multidrug resistance
IMN	intramedullary nail
	

## Code availability

No custom software or original analysis code was developed for this study. Statistical analyses were performed using IBM SPSS Statistics version 25.0.
