# Supplementary material for: Multi-regional debridement and staged reconstruction for fracture-related infection after intramedullary nailing
Source: J Bone Jt Infect. 2026 Oct 29;11(5):655–65. doi: 10.5194/jbji-11-655-2026 (PMC13723046; doi:10.5194/jbji-11-655-2026)
Supplement: Supplementary file 1 [file jbji-11-655-2026-supplement.pdf]

Supplement of J. Bone Joint Infect., 11, 655–665, 2026
https://doi.org/10.5194/jbji-11-655-2026-supplement
© Author(s) 2026. CC BY 4.0 License.

Journal of Bone and Joint Infection
Open Access

Supplement of

Multi-regional debridement and staged reconstruction for fracture-related infection after intramedullary nailing

Xingyu Chen et al.

Correspondence to: Yueliang Zhu (zhu123@zju.edu.cn), Muguo Song (ansen82@163.com), and Jian Shi (doctorshijian920@sina.com)

The copyright of individual parts of the supplement might differ from the article licence.

Table S1. Systemic antimicrobial strategy after Stage I

Microbiological category	n	Initial intravenous treatment	Continuation treatment	Total duration
MSSA	11	Susceptibility-directed intravenous therapy, mainly cefazolin, 14 days	Susceptibility-directed oral therapy, mainly levofloxacin plus rifampicin, 28 days	42 days
MRSA	4	Vancomycin, 14 days	Oral linezolid, 28 days	42 days
Enterococcus faecalis	3	Susceptibility-directed intravenous therapy; ampicillin was mainly used for susceptible isolates, 14 days	Susceptibility-directed continuation therapy; oral amoxicillin was mainly used for susceptible isolates, whereas intravenous therapy was continued when no active oral option was available, 28 days	42 days
Acinetobacter baumannii	3	Susceptibility-directed intravenous therapy, mainly ampicillin-sulbactam, 14 days	Susceptibility-directed continuation therapy; oral minocycline was mainly used when the isolate was susceptible and an active oral option was available; otherwise, intravenous therapy was continued, 28 days	42 days
Pseudomonas aeruginosa	3	Susceptibility-directed antipseudomonal intravenous therapy, mainly piperacillin-tazobactam, 14 days	Susceptibility-directed continuation therapy; oral ciprofloxacin was mainly used when the isolate was fluoroquinolone-susceptible and an active oral option was available; otherwise, intravenous therapy was continued, 28 days	42 days
Escherichia coli	4	Susceptibility-directed intravenous therapy, mainly ceftriaxone, 14 days	Susceptibility-directed continuation therapy; oral ciprofloxacin was mainly used when the isolate was fluoroquinolone-susceptible and an active oral option was available; otherwise, intravenous therapy was continued, 28 days	42 days
Klebsiella pneumoniae	2	Susceptibility-directed intravenous therapy; ceftriaxone was used in one susceptible isolate and ceftazidime-avibactam in one resistant isolate, 14 days	Susceptibility-directed continuation therapy; oral ciprofloxacin was mainly used when the isolate was fluoroquinolone-susceptible and an active oral option was available; otherwise, intravenous therapy was continued, 28 days	42 days
Culture-negative	17	Ceftriaxone 2 g intravenously once daily, 14 days	Levofloxacin 500 mg orally once daily plus rifampicin 450 mg orally once daily, 28 days	42 days

Note: Antimicrobial treatment in culture-positive cases was individualized according to the identified organism, susceptibility profile, patient tolerance, and the availability of an active oral agent. The agents listed represent the principal regimens

identifiable from the retrospective records and should not be interpreted as uniform protocols applied to every patient within each microbiological category. When no active oral agent was available or the isolate was resistant to available oral agents, susceptibility-directed intravenous therapy was continued during the continuation phase to complete the total 42-day course. Following repeat debridement, both affected patients restarted a complete 42-day systemic antimicrobial course. Patients proceeding to definitive Stage II reconstruction without evidence of active infection received routine surgical prophylaxis with a single pre-incision dose of cefazolin; no postoperative therapeutic antimicrobial course was administered. MSSA, methicillin-sensitive Staphylococcus aureus; MRSA, methicillin-resistant Staphylococcus aureus.

Table S2. Outcomes stratified by microbiological category

Microbiological category	No. of patients	Confirmed interstage infection, n	Confirmed post-reconstruction recurrence, n
Gram-positive infection	18	1	0
Gram-negative infection	12	0	0
Culture-negative infection	17	1	0
Total	47	2	0

Note: *Microbiological categories were based on the available deep-tissue culture results. One culture-negative patient underwent repeat debridement after histopathologically confirmed persistent infection was identified at the planned Stage II procedure. Both interstage events were classified as confirmed persistent infection and were not counted as post-reconstruction recurrence.*

Table S3. Stage I diagnostic sampling and laboratory procedures

Item	Result
Deep specimens per patient, mean ± SD	4.34 ± 0.96
Deep specimens per patient, median (IQR)	5 (4–5)
Range of specimen number	2–5
Two specimens obtained, n	4
Three specimens obtained, n	4
Four specimens obtained, n	11
Five specimens obtained, n	28
Deep soft-tissue specimen obtained, n	47
Lesional bone specimen obtained, n	47
Multi-site deep soft-tissue sampling, n	16
Specimens placed in separate sterile containers, n	47
Agar-based culture performed, n	47
Microbiological incubator used, n	47
Aerobic culture performed, n	47
Aerobic incubation duration	5 days
Anaerobic culture performed, n	17
Anaerobic incubation duration	14 days
Histopathological examination performed, n	47

Note: *IQR, interquartile range; SD, standard deviation. All specimens were obtained before empirical postoperative antimicrobial therapy was initiated. In the 17 ultimately culture-negative cases, anaerobic cultures were maintained for 14 days.*
